# How to Enhance the Diagnosis of Early Stages of Chronic Obstructive Pulmonary Disease (COPD)? The Role of Mobile Spirometry in COPD Screening and Diagnosis—A Systematic Review

**DOI:** 10.3390/arm92020018

**Published:** 2024-03-27

**Authors:** Piotr Jankowski, Katarzyna Mycroft, Katarzyna Górska, Piotr Korczyński, Rafał Krenke

**Affiliations:** Department of Internal Medicine, Pulmonary Diseases and Allergy, Medical University of Warsaw, 02-097 Warsaw, Poland; jankowskipiotr91@gmail.com (P.J.);

**Keywords:** COPD, spirometry, portable spirometer

## Abstract

**Highlights:**

**What are the main findings?**

- Portable spirometers are only slightly less efficient in diagnosing COPD than traditional spirometers
- The highest COPD prevalence was demonstrated when well-selected high-risk patients were tested

**What is the implication of the main finding?**

- Portable spirometers are useful in the early diagnosis of COPD
- Portable spirometers enable bedside COPD diagnosis

**Abstract:**

COPD is the third leading cause of death worldwide. Its diagnosis can be made with spirometry, which is underused due to its limited accessibility. Portable spirometry holds promise for enhancing the efficacy of COPD diagnoses. The study aimed to estimate COPD prevalence diagnosed with a portable spirometer in high-risk patients and compare it with COPD prevalence based on data from conventional, on-site spirometry. We also evaluated the strategy of a proactive approach to identify COPD in high-risk individuals. We conducted a systematic review of original studies on COPD targeted screening and diagnosis with portable and conventional spirometers selected from 8496 publications initially found in three databases: Cochrane, PubMed, and Embase. The inclusion criteria were met by 28 studies. COPD prevalence evaluated with the use of portable spirometers reached 20.27% and was lower compared to that estimated with the use of conventional spirometers (24.67%). In 11 included studies, postbronchodilator tests were performed with portable spirometers, which enabled a bedside COPD diagnosis. Portable spirometers can be successfully used in COPD targeted screening and diagnosis and thus enhance the detection of COPD at early stages.

## 1. Introduction

Chronic obstructive pulmonary disease (COPD) is highly prevalent; it is estimated that its affects around 7.6–10.3% of the worldwide population aged 30–79 years [1]. Although in early stages, the disease is mildly symptomatic [2] and approximately 80% of the affected subjects are unaware of its presence [3], COPD is a progressive and fatal disease. In 2019, COPD was the third leading cause of death worldwide [4].

A COPD diagnosis can be made in subjects with a significant exposure to risk factors (e.g., cigarette smoke) when fixed airway obstruction in spirometry is found [5]. Although spirometry is easy and inexpensive, it is largely underused [6]. This is probably due to the limited accessibility to office spirometry, lack of properly trained personnel, and physicians’ fear of potential misinterpretation. Moreover, patients with COPD are a psychologically difficult population, reluctant to undergo appropriate diagnostics. This group is often unwilling to seek medical assistance; difficulty in traveling to health care centers may also be a contributing factor [7]. On the other hand, the timely diagnosis and treatment of COPD are crucial in slowing down the progression of the disease, given that the decline in the forced expiratory volume in the first second (FEV_1_) tends to be more significant in the initial stages of the condition. [8]. Exacerbations of COPD can also manifest in its early stages, potentially resulting in hospitalization and increasing the risk of mortality [9]. Consequently, identifying and managing individuals with COPD, particularly in its early phases, can result in enhancements to their overall health conditions [10].

There are many different spirometers, ranging from advanced conventional devices used in well-equipped pulmonary function test laboratories in tertiary hospitals, to small-size spirometers used in primary care settings (most often desktop spirometers). For several years, portable easy-to-use spirometers have been available on the market. Importantly, the accuracy of such devices has been proven to be comparable to that of spirometers used in pulmonary function testing laboratories [11]. 

Small spirometers that can be connected to a smartphone via Bluetooth seem to bring significant technological progress to modern pulmonology. Nowadays, portable spirometers are used at patients’ homes to assess the control of asthma [12]. Portable spirometry can also be performed and supervised remotely in cystic fibrosis patients to monitor disease progression [13,14]. Other examples of the use of small spirometers also include spirometry at home to assess the efficacy of pirfenidone in patients with lung interstitial diseases [15] or the early detection of pulmonary complications in an allogeneic hematopoietic cell transplant recipients [16].

Portable spirometers can also be used in COPD diagnosis and follow-up. Previous articles that have examined the use of portable spirometers in patients with COPD focused mainly on the reliability of baseline spirometry and their use in population screening and targeted screening actions [17]. Some publications also assessed the possibility of using devices other than spirometers in COPD screening, e.g., the COPD-6 device [18] or a handheld expiratory flow meter [19].

Many past studies did not specify the tested populations, failing to differentiate among screening in asymptomatic patients at risk, true population health screening, and testing symptomatic individuals (diagnostic spirometry). In our work, we aimed to distinguish studies that focused on specific subpopulations of patients. Future publications concerning the effectiveness of portable spirometry should strive to distinguish the device’s effectiveness in screening actions in the general population, as well as in diagnostic spirometry.

There is only scarce data on the true diagnostic capabilities of portable spirometers. Thus, the authors decided to conduct a systematic review of the available articles that concerned COPD diagnosis in patients at risk using various types of spirometers, including portable devices. 

The specific objectives of this systematic review were:

1. To estimate COPD prevalence diagnosed with a portable spirometer in high-risk patients and compare it with the disease prevalence reported in the studies that used conventional spirometers;
2. To evaluate strategies of proactive approaches to identify COPD in high-risk individuals.

## 2. Materials and Methods

### 2.1. General Study Design

This was a systematic review of previously published original papers. The authors followed the recommendations of the PRISMA 2020 [20]. The protocol of this systematic review was registered in the PROSPERO registry (ID CRD42022337420).

### 2.2. Definitions

#### 2.2.1. Types of Screening

In line with the recommendations of the U.S. Preventive Services Task Force, the routine screening of asymptomatic patients for COPD is not advised due to its ineffectiveness. The diagnosis of COPD is instead recommended for individuals presenting respiratory symptoms, such as chronic cough, sputum production, difficulty breathing, or wheezing [21]. This type of COPD testing, focused on symptomatic individuals, is referred to as diagnostic spirometry.

Only a subset of the publications included in our systematic review met the criteria for diagnostic spirometry. However, we limited our focus to publications concerning individuals at an increased risk of COPD, with a minimum cumulative cigarette smoke exposure of 10 pack-years. Consequently, all included papers adhere to the definition of targeted screening as adopted by the UK National Screening Committee [22].

#### 2.2.2. Types of Spirometers

Spirometers differ in size, ranging from compact devices that fit in a pocket and can be easily connected to a smartphone (hand-held spirometers), to slightly larger models suitable for placement on a desk (desktop spirometers), and and culminating in spirometers utilized within pulmonary function test laboratories (conventional spirometers). However, in our paperwork we decided to apply a purely practical approach, classifying spirometers into 2 groups:

1. Portable spirometers—small/pocket devices, easily moved from room to room (also desktop spirometers), often connected to a smartphone, which can measure basic spirometric parameters (at least FEV_1_, FVC, and FEV_1_/FVC).
2. Conventional spirometers: a certified spirometer used in the pulmonary test laboratory that cannot be moved easily, usually used in the same office.

We believe that this this categorization enables the assessment of the feasibility of spirometers that can be used in active COPD diagnosis even outside healthcare facilities, as part of various spirometry initiatives.

### 2.3. Search Strategy

A systematic search of the literature was carried out to identify relevant, English language studies, published between 1958 and 7 December 2021. Pubmed, the Cochrane Central Register of Controlled Trials, and Embase databases were used as the source of the data. The keywords for our search included a combination of terms related to COPD (“obstructive pulmonary disease” [tiab] OR “obstructive lung disease” [tiab] OR “obstructive airway disease” [tiab] OR “airway or airflow obstruction” [tiab] OR “chronic bronchitis and pulmonary emphysema”), spirometry and screening (diagnosis [tiab] OR “case finding” [tiab] OR prevalence [tiab] OR “early detection” [tiab]) (Table S1). The wide search, including terms related not only to portable spirometers but to all of its types (including conventional spirometers), was performed in order to not miss any important study concerning targeted screening actions performed to detect COPD.

The reference lists of the selected articles were subjected to a hand search to identify additional articles.

### 2.4. Selection Criteria

Studies were eligible for inclusion if they met the PICOS criteria, namely the following:

1. Population: subjects aged ≥ 35 years with a history of smoking (≥10 pack-years).
2. Intervention: baseline spirometry performed with a portable device/conventional spirometer and a postbronchodilator test performed to confirm the COPD diagnosis (defined as FEV_1_/FVC < 0.7 [5] or <LLN [23]).
3. Comparison: n/a.
4. Outcome: prevalence of irreversible airway obstruction (i.e., airflow limitation not reversible after inhaled bronchodilator).
5. Study design: cross-sectional/cohort studies.

We excluded studies published in languages other than English, as well as non-original papers.

### 2.5. Study Selection

**Figure 1:**
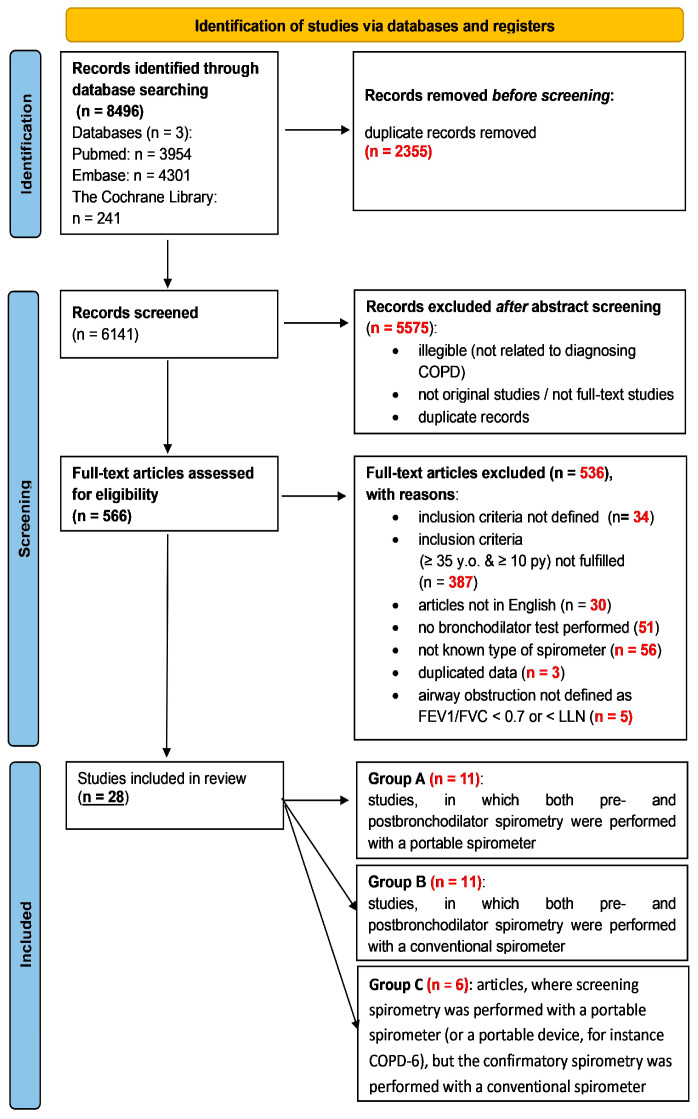
The process of screening and including articles for this systematic review; py—pack-years.

Relevant articles to be included in this review were identified and assessed independently by K.M. and P.J. All studies from the databases were screened by title and abstract. Irrelevant or duplicate articles were excluded, and all remaining articles were subjected to full-text screening. Differences between the reviewers in the inclusion of articles were resolved through discussion and consensus between all authors. Using the Joanna Briggs Institute critical appraisal tools for conducting JBI systematic reviews, studies underwent an evaluation to assess their methodological quality [24] (Table S2). Each study was assigned a score of either present (1) or absent (0), which was then aggregated to determine a final value. A significant risk of bias was identified when the percentage of positive responses was 49% or less. A moderate risk of bias was indicated when the percentage ranged between 50% and 69%. Conversely, a low risk of bias was detected when the percentage of positive responses exceeded 70%. Both reviewers (K.M. and P.J.) achieved consensus on the quality assessment outcomes through discussion. Figure 1 depicts the process of screening and including articles and lists the reasons for excluding articles. In the case of articles including no information about the spirometer’s type, an email was sent to the corresponding author with request to deliver the needed data. When no answer was received an article was excluded.

During the study selection, we observed that screening strategies could be categorized into three groups based on the type of spirometer employed (see: Data extraction). Studies included in the review, depending on the adopted strategy, are listed in Table 1, Table 2 and Table 3.

The whole process is pictured in the PRISMA flow diagram (Figure 1).

### 2.6. Data Extraction 

The following information was extracted from the included studies by two authors: (1) authors and year of publication; (2) country/region where the study was conducted; (3) spirometer type (portable or conventional); (4) number of participants; (5) inclusion criteria (6) baseline participants’ characteristics; (7) results, % of newly detected COPD cases (Table 1, Table 2 and Table 3). 

**Figure 2:**
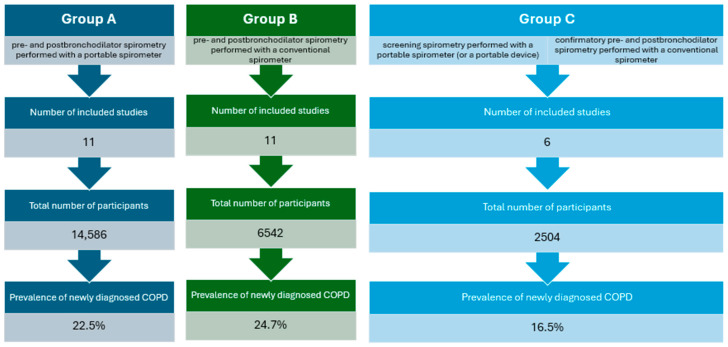
Comparison of distinguished publication groups.

In total, 28 studies were taken into consideration. The selected articles were classified into 3 categories depending on the type of the spirometer used to screen for and to diagnose COPD (Figure 2):

1. Studies in which both pre- and post-bronchodilator spirometry were performed with a portable spirometer (Group A, Table 1);
2. Studies in which both pre- and post-bronchodilator spirometry were performed with a conventional spirometer (Group B, Table 2);
3. Articles in which baseline spirometry was performed with a portable spirometer (or a portable device, for instance COPD-6), but the confirmatory spirometry was performed with a conventional spirometer (Group C, Table 3).

In one study [34], both portable and conventional spirometers were used to screen for COPD, and hence, we included the data from this study in two groups: A and B. In another paper [40], two different subgroups of patients were included (with and without respiratory symptoms), so we analyzed these subgroups separately. 

### 2.7. Statistical Analysis

Statistical analysis was performed using MedCalc^®^ Statistical Software version 20.218 (MedCalc Software Ltd., Ostend, Belgium; https://www.medcalc.org; 5 Januray 2024). A *p*-value of <0.05 was considered statistically significant. The primary outcome was the diagnostic yield with the 95% confidence interval (CI), which was calculated by dividing the number of successful diagnoses by the percentage of newly diagnosed COPD cases. Study heterogeneity was assessed using the Cochran Q test (χ^2^ test) and quantified based on the I^2^ index [51]. Statistical heterogeneity was indicated in cases of *p* < 0.01 with a χ^2^ test, and an I^2^ index value of >50% was considered significant heterogeneity [52]. Random-effect models with the inverse variance method were applied to reflect the variability of effect sizes among included studies with diversity in adjunctive modalities [53]. Publication bias was evaluated using funnel plot asymmetry based on both the Egger’s and Begg tests.

## 3. Results

### 3.1. Overview of Included Publications

The 28 publications included in our systematic review exhibited significant heterogeneity (Table 1, Table 2 and Table 3). Beyond the three categories highlighted according to the type of spirometer used, the included publications differed in terms of patients’ symptoms and the setting where the spirometry was performed.

#### 3.1.1. Risk of Bias

The majority of publications demonstrated a low risk of bias, indicating a high percentage of positive responses to the questions in the JBI tool (Table S2). In 6 out of 28 included studies, the risk of bias was evaluated as moderate. The risk of bias reached 50% in only one of the included papers [36], where the study subjects and the setting were not described in detail, and the outcomes were not measured in a valid manner.

#### 3.1.2. Symptomatic Patients

Out of the 28 selected publications, 12 studies focused on the diagnostic testing of COPD (Table S3). In these studies, patients with at least one respiratory symptom were eligible for spirometry. In the group of publications where respiratory symptoms were one of the inclusion criteria, there were four studies that used portable spirometers [25,30,31,34], six studies that used conventional spirometers [34,37,39,40,42,43], and two studies where the initial spirometry was performed using a portable spirometer or COPD-device and confirmed using a conventional spirometer [47,50].

In most publications, participants completed the COPD Assessment Test (CAT), the Modified Medical Research Council Dyspnea Scale (mMRC), or custom questionnaires, including, age, sex, smoking status, and the presence of respiratory symptoms, such as cough, phlegm, wheezing, and shortness of breath. In most cases, an individual was considered symptomatic if they exhibited at least one of the aforementioned symptoms. In some publications, the criterion regarding symptoms was more precise, as in the case of the paper by Yawn [37], in which patients were recruited for the study based on self-reported symptoms of chronic bronchitis, defined as the presence of a productive cough for at least three consecutive months in each of two successive years.

#### 3.1.3. Setting

The heterogeneity of the publications included in our systematic review is also evident in the setting where COPD targeted screening was conducted. In the majority of the studies, spirometry was performed in primary care settings. For some publications, volunteers were invited to undergo pulmonary function testing at a hospital. One described study was partially conducted at a railway station [48]. Two of the studies included by us were conducted on hospitalized patients [33,34].

### 3.2. Portable Spirometers

**Figure 3:**
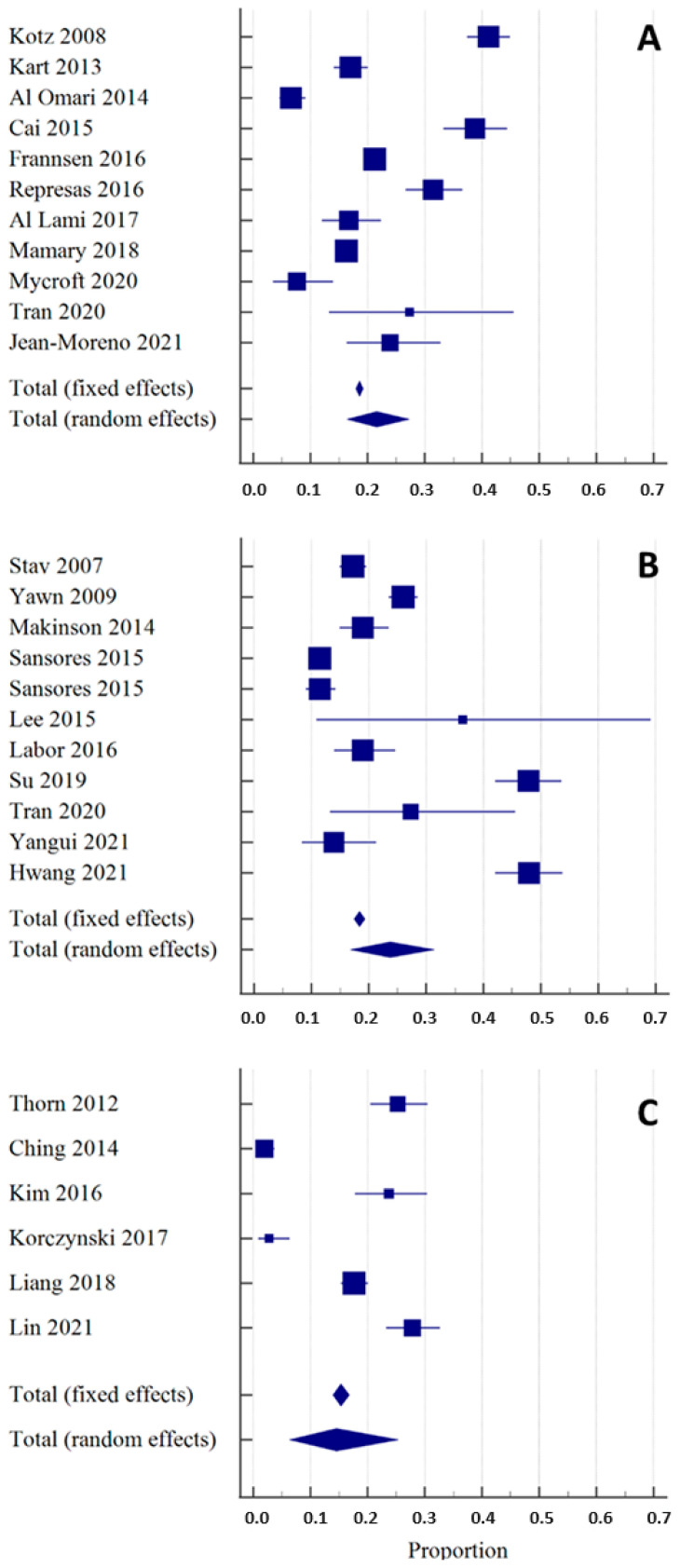
Overall diagnostic yield of spirometers in the detection of new COPD cases shown as the weighted summary proportion (expressed as a percentage), with their 95% CIs, found in the individual studies included in the systematic review [25,26,27,28,29,30,31,32,33,34,35,36,37,38,39,40,41,42,43,44,45,46,47,48,49,50]. The size of the square corresponds to the size of the studied population. Section (**A**): publications that used portable spirometers. Section (**B**): publications that used conventional spirometers. Section (**C**): publications that used a double-step strategy, using a portable spirometer or a COPD-6 device for baseline spirometry and a conventional spirometer for the postbronchodilator test.

Eleven studies using portable spirometers in the COPD-targeted -screening process met the predefined criteria (Group A, Table 1) [25,26,27,28,29,30,31,32,33,34,35]. In all selected articles, a portable spirometer was used both for the baseline spirometry and in the postbronchodilator testing. The included studies differed in the study population’s size, airway obstruction criteria, and setting. The percentage of newly found COPD cases among studies using portable spirometers ranged from 6.6% to 41%, with the proportion (random effects) of 21.5% (95% CI 16.4–27.2) (Figure 3A). There was substantial heterogeneity across studies with I2 97.3% (95%CI 96.6–98.1), *p* < 0.0001. Egger’s and Begg’s tests excluded publication bias with *p* = 0.34 and *p* = 0.81, respectively. Importantly, some of the selected articles included only subjects with respiratory symptoms [25,30,34]. In those studies, average COPD detection was higher (29.08%) than in the remaining studies (15.43).

The largest smoker populations were analyzed in two studies [29,32] that used the portable spirometer EasyOne (NDD Medical Technologies). The Canadian study by Mamary et al. [32] was conducted on the general smoker population (*n* = 8872) in the primary care setting and reported an incidence of newly diagnosed COPD of 16.3%; 20% of the previously undetected COPD patients had been already diagnosed with asthma. A higher COPD incidence (21.2%) was reached in the European study by Frannsen and colleagues. In this study, a pulmonary function test was performed on patients (*n* = 2730) burdened with ischemic heart disease (IHD) attending cardiology outpatient clinics. Only patients with an established IHD diagnosis were included, irrespective of their reported pulmonary symptoms.

The efficacy of spirometry perfomed with a portable device in COPD targeted screening was also confirmed in a study conducted in patients with severe mental disorders, such as schizophrenia or bipolar disease [35]. Only patients without a prior lung disease diagnosis were included. COPD was detected in almost one of four patients studied, with the vast majority (78%) presenting with moderate or severe disease stages (GOLD II and III). According to the study results, in most cases (85%), COPD was clinically indolent.

In two of the included studies, baseline spirometry was performed for hospitalized smokers at bedside [33,34]. The complete COPD diagnostic process (both pre- and postbronchodilator spirometry) proved successful without the need to transport the patients to a pulmonary test laboratory. Small spirometers (MicroLab and AioCare) were used with a COPD detection rate of 27% (patients hospitalized due to productive cough and dyspnea [25]) and 7.6% (inpatients of pulmonary and cardiology departments meeting the criteria for age and smoking history [33]). Hospitalized smokers from these studies were the oldest population tested with the use of a portable spirometer among studies included in our review, with the average patient age above 65 years. 

The other spirometry targeted screening action performed in the hospital setting (however not among inpatients) was performed in Turkey [26]. Volunteers aged above 40 years with a smoking history of more than 10 pack-years who visited the hospital for any reason had spirometry performed in the hospital’s garden with the use of a portable spirometer; 17% of the participants were newly diagnosed with COPD. 

The highest incidence (>30% of patients) of newly diagnosed COPD was achieved in studies conducted in the primary care setting [25,28,30]. The studies by Kotz and colleagues [25] and Represas-Represas [30] enrolled only smokers with respiratory symptoms. In the study performed in China [27], older (>50 years of age) smokers with increased exposure to tobacco smoke (>20 py) were recruited, which could possibly result in a higher positive COPD prevalence (38.8%). It is also not clear whether patients with previously detected and treated COPD were excluded from the analysis. 

The youngest smokers (of at least 35 years of age) were invited to the COPD targeted screening programs in two studies performed in Middle East countries [27,31]. Both studies were conducted in the primary care setting with the use of handheld spirometers (Discovery 2 and FlowscreenCT). In the study by Al Lami et al., spirometry was performed only among smokers with respiratory symptoms, which can explain the higher prevalence of newly diagnosed COPD compared with the second above-mentioned study (16.7% vs. 6.6%). 

### 3.3. Conventional Spirometers

We identified 11 studies that utilized conventional spirometers for conducting COPD targeted screening actions (Group B, Table 2) [34,36,40,41,42,43,44]. 

The detection of new COPD cases among studies using conventional spirometers ranged from 11.4% to 47.8%, with the proportion (random effects) of 23.7% (95% CI 16.8–31.4) (Figure 3B). There was substantial heterogeneity with I2 97.5% (95% CI 96.7–98.2), *p* < 0.0001. Egger’s and Begg’s test excluded publication bias with *p* = 0.17 and *p* = 0.31, respectively.

As anticipated, the highest incidence of newly diagnosed COPD was achieved in studies that included only subjects presenting with respiratory symptoms [37,39,42,43]. In two of the above studies [42,43], nearly half of the patients (47.9%) were newly diagnosed with COPD. Both studies were performed at hospitals. The study by Su et al. [42] was conducted in Taiwan on patients referred to hospitals from pulmonary outpatient clinics. Relevantly, the investigated group consisted of well-selected patients with a history of a minimum of 20 pack-years and the average age of above 70 years. In the study conducted by Hwang et al. [43], COPD targeted screening was performed in the South Korean tertiary hospitals on patients presenting with respiratory symptoms, such as dyspnea or a productive cough. 

A high rate of newly detected COPD cases (36.4%) was also achieved in the study by Lee [39], in which a very small population of elderly patients (>75 yrs.) with a history of a minimum of 20 pack-years was recruited. This study was performed in the primary care setting. 

Two of 11 studies, which used laboratory spirometers for COPD targeted screening action, were conducted on a specific group of smokers—infected with HIV [38] and with ischemic heart disease [44]. The higher incidence of newly recognized COPD cases was achieved in the study with an inclusion criterion of a more relevant smoking history (min. 20 pack-years in the study by Makinson et al. [38] vs. 10 pack-years in the study by Yangui et al. [44]).

In our analysis of COPD targeted screening actions utilizing conventional spirometers, we treated the article by Sansores et al. [40] as comprising two independent studies. This approach was applied because the study examined two distinct patient subpopulations: individuals with respiratory symptoms and those without. By implementing more precise inclusion criteria, the study revealed a significant increase in the incidence of newly detected COPD cases (11.4% vs. 5.7%).

### 3.4. Baseline Testing with a Portable/COPD-6 Device, Confirmatory Spirometry with a Conventional Spirometer

We found six studies in which a COPD diagnostics were performed in two steps with the use of two different spirometer types (Table 3) [45,46,47,48,49,50]. In all of these studies, the first step the baseline spirometry was either performed with a portable spirometer [48,50] or with the use of a COPD-6 device [45,46,47,49]. Confirmatory spirometry leading to a COPD diagnosis was performed with a conventional spirometer.

The detection of new COPD cases among studies using both types of spirometers ranged from 1.9% to 27.8%, with the proportion (random effects) of 14.6% (95% CI 6.4–25.3) (Figure 3C). There was substantial heterogeneity across studies with I2 97.7% (95% CI 96.5–98.5), *p* < 0.0001 Egger’s and Begg’s test excluded publication bias with *p* = 0.82 and *p* = 0.85, respectively.

As in the previously described groups, the highest incidence of newly detected COPD was found in the studies, which recruited patients with at least one respiratory symptom (27.8%) [50]. In this study, performed in the primary care setting, all eligible participants underwent the targeted screening spirometry with the hand-held spirometer Spirobank Smart and, regardless of its results, the confirmatory postbronchodilator spirometry with the diagnostic spirometer. 

A similar approach was applied in the study by Kim et al. [47]. Only subjects complaining of respiratory symptoms were enrolled. Regardless of the result of FEV_1_/FEV_6_ measured with the COPD-6 device in the primary care setting, all study participants were then reffered for laboratory spirometry conducted in tertiary hospitals. The COPD detection rate was 23.7%. 

Also in the study by Thorn et al. [45], irrespective of the results of the initial test performed using the COPD-6 device, all participants underwent confirmatory spirometry with a COPD detection rate of 25.2%.

Other targeted screening methods were applied in the studies performed in Poland [48] and in Malaysia [46]. The first study was conducted in an unusual setting. Medical students recruited smoking passengers (min. 10 pack-years) at a railway station. A baseline spirometry was performed with the use of a portable spirometer MicroLab 3500 (Care Fusion). Among all participants, only those with airway obstruction were encouraged to undergo stationary spirometry in a pulmonary department. The low incidence of newly detected COPD patients (2.8%) was probably due to the very low response rate—only 15 out of 37 participants with airflow obstruction came forward for confirmatory spirometry.

In the study conducted in a primary care setting in Malaysia [46], an initial test was performed with a COPD-6 device. Only subjects with the suspicion of airflow limitation (FEV_1_/FEV_6_ < 0.75) were asked to return for formal spirometry testing. Similarly to the study by Korczyński et al. [48], only a few participants attended a confirmatory test, resulting in a low incidence of newly detected COPD (1.9%).

A higher rate of successful COPD targeted screening was achieved in the study by Liang et al. [49] (17.6%), where confirmatory spirometry was performed at the same place (general practice clinics in Australia) directly succeeding the baseline test (conducted with a COPD-6 device). 

### 3.5. Incidence of COPD

**Figure 4:**
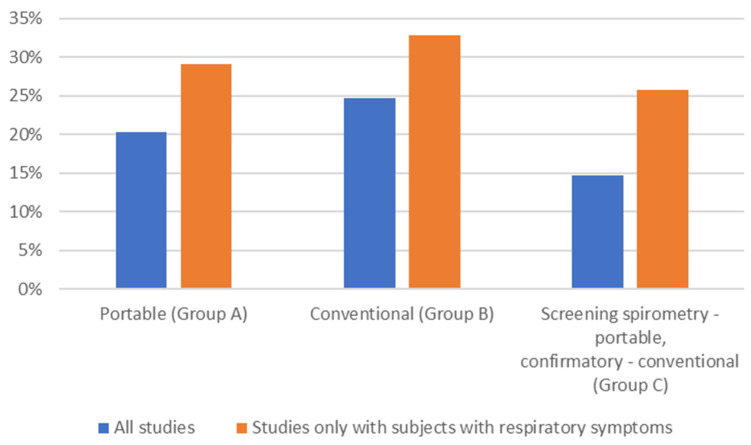
Incidence of newly detected COPD cases with the use of different spirometer types (average; %). All studies: blue; studies that included only subjects with respiratory symptoms: orange.

The incidence of newly detected COPD cases with the use of different spirometer types is shown in Figure 4. In all groups (A–C), the incidence of newly diagnosed COPD cases was higher in studies in which only subjects presenting with respiratory symptoms were included.

## 4. Discussion

### 4.1. Principal Findings

To our knowledge, this is the first systematic review to assess the feasibility of using small, handheld, or pocket-sized spirometers for COPD targeted screening and detection, in addition to comparing the results of portable and conventional spirometry targeted screening actions.

Our study shows that portable spirometric tools are slightly less efective in the COPD targeted screening actions as traditional, laboratory spirometers. This type of spirometer is not only useful for pre-bronchodilator testing, but can be considered a reliable tool when performing postbronchodilator spirometry and thus in confirming an irreversible airflow limitation, which in smokers, is equivalent to a COPD diagnosis. In a few studies analyzed in our systematic review, a handheld, mobile-phone linked spirometer proved advantageous for COPD targeted screening at the bedside of hospitalized patients not capable of being transported to a pulmonary function testing laboratory.

According to this systematic review, diagnostic COPD spirometry is more effective than screening asymptomatic individuals. A higher rate of COPD diagnoses was achieved in studies that included older participants with respiratory symptoms and a history of many pack-years.

Moreover, if a targeted screening action is to be successful, the postbronchodilator test has to be performed on site, directly succeeding the baseline test. Among over 500 excluded studies, the lack of a postbronchodilator test was the third cause of exclusion after not meeting the age criterion and the lack of information about the type of spirometer.

### 4.2. Methodology

A precise methodology has to be established to obtain a good-quality study and reliable evidence. Our aim was to find as many studies in which portable spirometry was used in a COPD targeted screening action as possible. Our general approach was to search only for studies that ended in a disease diagnosis. We are convinced that we achieved this goal. Secondly, we aimed to make our study clear and easy to read. To achieve this, we used a PRISMA 2020 flow diagram added as a methodology figure. Furthermore, we rejected a significant number of studies (over 8450). It is well-known that a proper review needs to be double-checked. Therefore, two of the authors performed the rejection process twice separately to be sure that none of the appealing studies had been missed. 

### 4.3. Strong Points of the Study

To our knowledge, the current study is the first systematic review to examine the feasibility of spirometry perfomed with portable spirometers in COPD targeted screening. It has to be emphasized that all studies included in our review led not only to the detection of airflow limitation, but directly to a COPD diagnosis, as in all found studies a post-bronchodilator test was performed. Beyond the scientific value, the findings of our research have clinical importance, as we proved that with the help of a portable spirometer, a proper COPD diagnosis can be made and an appropriate treatment can be launched. It is widely known that treating patients with COPD, especially in the early stages, can lead to an improvement in their health status [10].

An additional advantage of our work is the fact that we have applied very strict inclusion criteria. Only publications confirming persistent obstruction were considered as indicating COPD, with the exclusion of studies where individuals with asthma were part of the study population.

**Figure 5:**
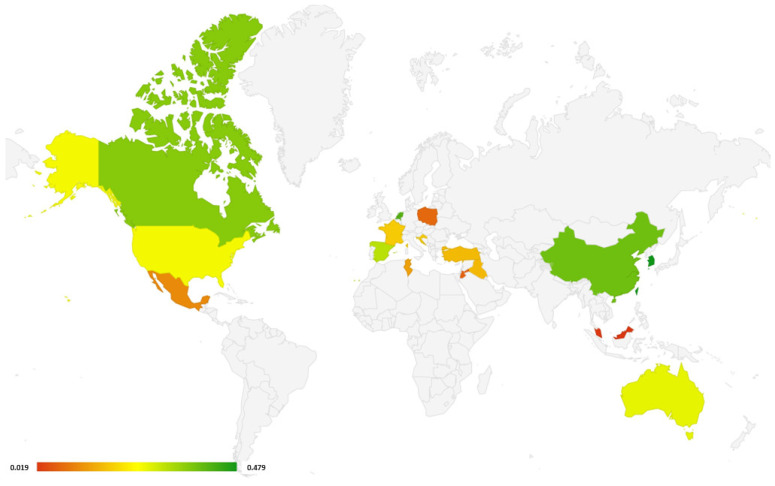
World map with countries, where studies included in this systematic review were performed (all types of spirometers). Different colors depict the % of newly diagnosed COPD (green color: high rate of newly detected COPD cases; red color: low rate). If in one country more than one study was performed, the study with the highest rate of newly detected COPD cases was selected.

Another advantage of this systematic review is that studies from all over the world have been selected (Figure 5). According to the results of the study, the countries of North America and the Far East have the greatest effectiveness in diagnosing COPD in spirometry actions.

### 4.4. Potential Confounding Aspects and Limitations of the Study

This systematic review has some limitations. Although the number of records identified through the database search was considerable, the rigorous inclusion criteria to select papers evaluating the use of a portable spirometer for the diagnosis of COPD resulted in relatively few matching papers ultimately being included in this systematic review remaining. Firstly, we excluded studies in which a postbronchodilator test was not performed. We also excluded papers in which it was not clear what type of spirometer was used for the COPD targeted screening action and the corresponding author of the paper did not provide a response to our inquiry. Secondly, in two included studies from two different groups (A and C) [28,45], it was not known if patients with previously diagnosed COPD were excluded from the studied population. In this case, it may have led to an overstatement of newly detected COPD cases and the effectiveness of COPD targeted screening actions.

Thirdly, despite our initial exclusion criteria of publications where individuals with previously diagnosed asthma were part of the study population, it is possible that some smokers with persistent obstruction were actually suffering from this condition. It is clear that in distinguishing between COPD and asthma, clinical symptoms are important, not just the results of additional tests, such as spirometry. Unfortunately, clinical symptoms were not reported in all included publications, which could result in an overestimation of COPD prevalence.

Another limitation of our study was the fact that we decided to include studies that were not conducted with the help of a portable spirometer, but a COPD-6 device. This tool is not capable of measuring the FEV1/FVC, but can measure an alternative FEV1/FEV6. However, in studies included in group C, a confirmatory spirometry leading to COPD detection was always conducted with a confirmatory spirometer. Lastly, the included studies exhibited significant heterogeneity. Selected publications differed, among other factors, in the criteria used for patient selection (e.g., various questionnaires assessing patients’ symptoms). Only a few analyzed studies assessed the quality of spirometry tests performed. Furthermore, included studies employed different spirometry criteria for diagnosing COPD. The most commonly used criterion was the one recommended by GOLD (FEV1/FVC < 0.70 in the postbronchodilator test) [5], followed by <LLN [24]. The included papers also varied in terms of the adopted technical standards for conducting spirometry tests and spirometry reference values. Although we are aware that reference values likely varied as well, partly due to the time span and geographic scope of the analyzed publications (2007–2021), almost none of the cited publications provided information on this topic.

Unfortunately, not all included papers provided data on the coexistence of other chronic diseases, such as diabetes or arterial hypertension. Only in isolated cases did the included publications contain data on the coexistence of congestive heart failure, which undoubtedly could have affected the results of spirometry.

The analyzed studies also do not include data on the time and quality of training of individuals performing spirometry, both for portable spirometers, as well as conventional ones, which could have influenced the results. According to the results of the Chinese meta-analysis [14], the effectiveness of portable spirometers in diagnosing airway obstruction, among other factors, depended on the proper technical execution of spirometry. As demonstrated in previous publications [54]; the percentage of correctly performed spirometry tests leading to the diagnosis of airway obstruction using a portable spirometer increased with the experience of the person conducting the test and the duration of training. Future analyses comparing the effectiveness of using portable and conventional spirometers in diagnosing COPD should be designed in such a way that the individuals conducting the tests are properly and uniformly trained. This will result in the achievement of reproducible tests and likely in a higher prevalence of COPD.

### 4.5. Comparison with Other Studies

Our review provides the first comprehensive summary of up-to-date evidence on the feasibility of portable spirometers for the targeted screening and, simultaneously, the diagnosis of COPD. This systematic review included 28 articles, of which 11 concerned COPD targeted screening with the use of portable spirometers. To compare, in their meta-analysis, Zhou et al. [14] identified 31 studies that systematically evaluated the diagnostic value of portable spirometers for detecting COPD. However, a different aim of this study was chosen and a different approach was applied, as in included articles, portable devices were used to determine airway obstruction, not necessarily a COPD diagnosis. Only in one study [25], which was also included in our systematic review, was a post-bronchodilator test conducted using a portable spirometer (Datospir 120, Sibelmed), which led to confirmed COPD diagnoses in over 31% of cases. The same study [30] was also found in the systematic review published in 2021 [55]. Also in this review, it was the only included study in which a portable spirometer was used to perform a post-BD spirometry. The other 12 identified articles measured the sensitivity and specificity of the portable devices (mainly COPD-6) in diagnosing airway obstruction, which were not used to conduct the post-BD test.

### 4.6. Benefits of Portable Spirometers

According to the results of our systematic review, the diagnostic value of portable spirometers for COPD targeted screening is slightly lower than that of conventional spirometers. As indicated above, this could be attributed to various factors, including significant heterogeneity among the included publications. However, the numerous advantages of the portable spirometer make its use in COPD testing worthy of further investigation, preferably with consideration given to the appropriate design of the study. Important advantages of portable spirometers include, among others, the ability to examine patients at their hospital bedside, as well as cost-effectiveness. Studies have shown that portable-spirometer-targeted screening is cost-saving in primary care patients presenting with respiratory symptoms compared with questionnaire screening and no screening [56].

### 4.7. Minimal Standards of Portable Spirometers

A portable spirometer should meet basic standards. Although it has been demonstrated that the FEV1/FEV6 can be regarded as a viable surrogate indicator for diagnosing COPD [14], a portable spirometer, according to its definition, must be able to measure at least FEV1, FVC, and FEV1/FVC. Portable spirometry also needs to be performed under strict quality control. To achieve consistent results, it is important to emphasize proper training for individuals conducting spirometry using portable devices. Spirometry performed using these devices should adhere to applicable technical standards and current reference norms.

## 5. Conclusions

Present studies suggest that portable spirometers are only slightly less efficient in diagnosing COPD compared to traditional spirometers. Future spirometry targeted screening for the diagnosis of COPD, in order to increase its effectiveness, should be considered in selected symptomatic subjects.

## Figures and Tables

**Table 1:** Studies conducted with a portable spirometer.

	First Author, Year of Publication	Country/ Region	Spirometer (Manufacturer)	Participants: n (%F)	Population (Prevalence of Comorbidities: DM%; HTN%)	Inclusion Criteria: Age; PY	Age, Years (Mean: SD)	PY (Mean: SD)	Setting	FEV_1_/FVC Cut off Value for Airway Obstruction	% Newly Diagnosed COPD
1	Kotz, 2008 [25]	The Netherlands	Vitalograph 2120 (Vitalograph)	676 (41.3)	GP (ND)	40–70; ≥10	52.3 (7.3)	40.4 (19.3)	ND	0.7	41.1 ^1^
2	Kart, 2013 [26]	Turkey	MIR A23 (MIR)	648 (61.9)	GP (ND)	>40; ≥10	48.3 (9)	ND	HS	0.7	17
3	Al Omari, 2014 [27]	Jordan	FlowscreenCT (eResearch Technology GmbH)	512 (0)	GP (ND)	>35; >10	48.3 (10.2)	42.7 (10–200) ^3^	PC	0.7	6.6
4	Cai, 2015 [28]	China	Portable	307 (8.8)	GP (ND)	>50; >20	61 (7)	37 (15)	HS	0.7	38.8 ^4^
5	Frannsen, 2016 [29]	Europe	EasyOne (Medical Technologies)	2730 (14.1)	IHD (25.3; 89.1)	≥40; ≥10	ND	ND	OSC	0.7	21.2
6	Represas-Represas, 2016 [30]	Spain	Datospir 120 (Sibelmed)	362 (38.1)	GP (ND)	>40; ≥10	55.4 (9.9)	35 (19.8)	PC/ PHA/ HS	0.7	31.5 ^1^
7	Al Lami, 2017 [31]	Iraq	Discovery 2 (Futuremed)	215 (ND)	GP (18.8; 37.8)	>35; >20	ND	ND	PC	0.7	16.7^1^
8	Mamary, 2018 [32]	USA	EasyOne (Medical Technologies)	8872 (45.6)	GP (ND)	45–80; ≥10	59.9 (9.1)	44.5 (25.1)	CSC	0.7	16.3
9	Mycroft, 2020 [33]	Poland	AioCare (HealthUp)	118 (33.1)	HO (22;92)	≥40; ≥10	66 (59–73) ^2^	30 (20–40) ^2^	HS	LLN	7.6
10	Tran, 2020 [34]	Australia	MicroLab	33 (42)	HO (21;58)	>40; >10	69.3 (6.8)	48.7 (24.2)	HS	0.7	27.2 ^1^
11	Jaen-Moreno, 2021 [35]	Spain	DatoSpir Touch Easy D (Sibelmed)	113 (ND)	MENT (13.4; 8.5)	40–70; ≥10	49.4 (6)	36.6 (18.1)	HS	0.7	23.9

CSC: clinical study center; DM: diabetes mellitus; GP: general population; HO: hospitalized; HS: hospital; HTN: hypertension; IHD: patients with ischemic heart disease; MENT: patients with severe mental illness; ND: no data available; OSC: outpatient specialty clinic; PC: primary care; PHA: pharmacy; PY: pack-years. ^1^ Studies in which respiratory symptoms were one of the subject’s inclusion criteria; ^2^ data are presented as the median (interquartile range) or ^3^ range; ^4^ not known if patients with previously diagnosed COPD were excluded.

**Table 2:** COPD diagnostic studies performed with conventional spirometers.

	First Author, Year of Publication	Country/ Region	Spirometer (Manufacturer)	Participants: n (%F)	Population (Prevalence of Comorbidities: DM%; HTN%)	Inclusion Criteria: Age; PY	Age, Years (Mean: SD)	PY (Mean: (SD)	Setting	FEV_1_/FVC Cut off Value for Airway Obstruction	% Newly Diagnosed COPD
1	Stav, 2007 [36]	Israel	Jaeger, CareFusion	1058 (25)	GP (ND)	45–75; ≥20	ND	ND	ND	0.7	17.2
2	Yawn, 2009 [37]	USA	Biomedical Systems Corporation	1201 (ND)	GP (ND;41%)	>40; ≥10	ND	ND	PC	≤0.7	26 ^1^
3	Makinson, 2014 [38]	France	laboratory spirometers	338 (17)	HIV (ND)	≥40; ≥20	50 (46–53) ^2^	30 (25–38) ^2^	HS	0.7	18.9
4	Lee, 2015 [39]	Canada	Winspiro (MIR)	11 (ND)	GP (22.9;51.2)	≥75; ≥20	ND	ND	PC	0.7	36.4 ^1^
5	Sansores, 2015 [40]	Mexico	Sensormedics	2324 (50.1)	GP (ND)	>40; ≥10	51.9 (10.5)	19.50 (10–33) ^2^	PC	LLN	11.4 ^1^
6	Sansores, 2015 [40]	Mexico	Sensormedics	637 (52)	GP (ND)	>40; ≥10	49.63 (11.3)	17.00 (8–28) ^2^	PC	LLN	5.7
7	Labor, 2016 [41]	Croatia	Jaeger, CareFusion	227 (50.6)	GP (ND)	40–65; ≥20	52.5 (6.8)	37.9 (17.4)	PC	0.7	18.9
8	Su, 2019 [42]	Taiwan	Spiro Medics system 2130 (SensorMedics)	301 (4.7)	PULMO (ND)	≥40; ≥20	70.7 (13.2)	45.4 (25.0)	HS	0.7	47.9 ^1^
9	Tran, 2020 [34]	Australia	HypAir Compact+ (Medisoft)	33 (42.4)	HO (21;58)	>40; >10	69.3 (6.8)	48.7 (24.2)	HS	0.7	27.2 ^1^
10	Hwang, 2021 [43]	South Korea	conventional spirometer	290 (ND)	GP (ND)	>40; >10	63.1 (11.0)	31.6 (20.0)	HS	0.7	47.9 ^1^
11	Yangui, 2021 [44]	Tunisia	COSMED Quark Series	122 (1.7)	IHD (55.7;46.7)	>40; ≥10	59.3 (9.5)	52.3 (28.3)	HS	0.7	13.9

DM: diabetes mellitus; GP: general population; HIV: HIV-infected patients; HS: hospital; HTN: hypertension; IHD: patients with ischemic heart disease; LLN, lower limit of normal; ND: no data available; PC: primary care; PULMO, patients from pulmonary outpatient clinics; PY, pack-years. ^1^ Studies in which respiratory symptoms were one of the subject’s inclusion criteria; ^2^ data are presented as the median (interquartile range).

**Table 3:** COPD diagnostic studies: baseline spirometry performed with a portable spirometer or COPD-6 device; confirmatory with a conventional spirometer.

	First Author, Year of Publication	Country/ Region	Device (Manufacturer) ^3^	Participants: n (%F)	Population (Prevalence of Comorbidities: DM%; HTN%)	Inclusion Criteria: Age, PY	PY (Mean: (SD)	Setting	FEV_1_/FVC Cut off Value for Airway Obstruction	% Newly Diagnosed COPD
1	Thorn, 2012 [45]	Sweden	COPD-6	305 (56.7)	GP (ND)	45–85; ≥15	30.3 (11.5)	PC	0.7	25.2 ^2^
2	Ching, 2014 [46]	Malaysia	COPD-6	416 (0.2)	GP (ND; 46.2)	≥40; ≥10	20.4 (18)	PC	0.75/0/7 ^4^	1.9
3	Kim, 2016 [47]	South Korea	COPD-6	190 (ND)	GP (17.8; 40)	>40; >10	28.5 (14.6)	PC	0.77/0.7 ^4^	23.7 ^1^
4	Korczyński, 2017 [48]	Poland	MicroLab 3500, CareFusion	178 (36.5)	GP (ND; 67)	>40; >10	28	RS	0.7	2.8
5	Liang, 2018 [49]	Australia	COPD-6	1045 (ND)	GP (ND)	≥40; ≥10	ND	PC	0.75/0.7 ^4^	17.6
6	Lin, 2021 [50]	Taiwan	Spirobank Smart	370 (5.7)	GP (ND)	≥40; ≥10	42.6 (28.3)	PC	0.7	27.8 ^1^

DM: diabetes mellitus; GP: general population; HTN: hypertension; ND: no data available; PC: primary care; PY: pack-years; RS: railway station. ^1^ Studies in which respiratory symptoms were one of the subject’s inclusion criteria; ^2^ not known if patients with previously diagnosed COPD were excluded; ^3^ postbronchodilator spirometry was performed with a conventional spirometer; ^4^ the first value shows the FEV_1_/FEV_6_ cut-off value for airway obstruction for the COPD-6 device, and the second value shows the FEV_1_/FVC cut-off value for airway obstruction for the conventional spirometer.

## Data Availability

All data generated or analyzed during this study are included in this article. Further enquiries can be directed to the corresponding author.

## Supplementary Materials

The following supporting information can be downloaded at: https://www.mdpi.com/article/10.3390/arm92020018/s1, Table S1: Search strategy for PubMed; Table S2: JBI assessment results of studies; Table S3: Publications addressing the diagnostic screening of COPD along with the characterization of patient inclusion criteria.
