# Supplementary material for: How to Enhance the Diagnosis of Early Stages of Chronic Obstructive Pulmonary Disease (COPD)? The Role of Mobile Spirometry in COPD Screening and Diagnosis—A Systematic Review
Source: Adv Respir Med. 2024 Mar 27;92(2):158–74. doi: 10.3390/arm92020018 (PMC11047510; doi:10.3390/arm92020018)
Supplement: Supplementary file 1 [file arm-92-00018-s001.zip › arm-2836977-supplementary.pdf]

advances in respiratory medicine

MDPI

Supplementary Materials

Table S1. Search strategy for PubMed.

No.	Concept, domain	Search term, query
1	Search terms for: spirometry (title/abstract/keywords)	spirometry[tiab] OR spirometer[tiab] OR spirometer[tiab] OR spirometers[tiab] OR spirometries[tiab] OR "pulmonary function test"[tiab] OR "pulmonary function tests"[tiab]
2	MeSH terms for: spirometry	"Spirometry"[Mesh]
3	Search terms for: screening (title/abstract/keywords)	screening[tiab] OR screenings[tiab] OR "case finding"[tiab] OR prevalence[tiab] OR "early detection"[tiab] OR "early identification"[tiab] OR diagnosis[tiab] OR diagnoses[tiab]
4	MeSH terms for: screening	"Mass Screening"[Mesh] OR "Diagnosis"[Mesh:NoExp] OR "Early Diagnosis"[Mesh:NoExp]
5	Search terms for: COPD (title/abstract/keywords)	COPD[tiab] OR "obstructive pulmonary disease"[tiab] OR "obstructive pulmonary diseases"[tiab] OR "obstructive lung disease"[tiab] OR "obstructive lung diseases"[tiab] OR "airway obstruction"[tiab] OR "airways obstruction"[tiab] OR "airway obstructions"[tiab] OR "airways obstructions"[tiab] OR "obstructive airway disease"[tiab] OR "obstructive airway diseases"[tiab] OR "airflow obstruction"[tiab] OR "airflow obstructions"[tiab] OR “pulmonary emphysema”[tiab] OR "pulmonary emphysemas"[tiab] OR "focal emphysema"[tiab] OR "focal emphysemas"[tiab] OR "panacinar emphysema"[tiab] OR "panacinar emphysemas"[tiab] OR "panlobular emphysema"[tiab] OR "panlobular emphysemas"[tiab] OR "centriacinar emphysema"[tiab] OR "centriacinar emphysemas"[tiab] OR "centrilobular emphysema"[tiab] OR "centrilobular emphysemas"[tiab] OR bronchitis[tiab]
6	MeSH terms for: COPD	"Pulmonary Disease, Chronic Obstructive"[Mesh:NoExp] OR "Pulmonary Emphysema"[Mesh] OR "Bronchitis, Chronic"[Mesh]
7	Combined search blocks	(#1 OR #2) AND (#3 OR #4) AND (#5 OR #6)
8	Search filter	NOT (review[pt] OR "systematic review"[pt] OR "case reports"[pt] OR "editorial"[pt] OR "letter"[pt] OR "comment"[pt]) NOT ("animals"[mesh] NOT "humans"[mesh])
	Total search, items found	7 AND 8, N= 3954

Table S2. JBI assessment results of studies.

First author, year of publication	1. Were the criteria for inclusion in the sample clearly defined ?	2. Were the study subjects and the setting described in detail?	3. Was the exposure measured in a valid and reliable way?	4. Were objective, standard criteria used for measurem ent of the condition ?	5. Were confounding factors identified?	6. Were strategies to deal with confoun ding factors stated?	7. Were the outcomes measured in a valid and reliable way?	8. Was appropriate statistical analysis used?	Total (% of of positive responses)	Risk of bias
Kotz, 2008 [25]	1	1	1	1	0	0	1	1	6/8 (75)	low
Kart, 2013 [26]	1	1	1	1	0	0	1	1	6/8 (75)	low
Al Omari, 2014 [27]	1	0	1	1	1	0	1	1	6/8 (75)	low
Cai, 2015 [28]	1	0	1	1	1	0	1	1	6/8 (75)	low
Frannsen, 2016 [29]	1	1	1	1	1	1	1	1	8/8 (100)	low
Represas-Represas, 2016 [30]	0	0	1	1	1	0	1	1	5/8 (62.5)	moderate
Al Lami, 2017 [31]	1	1	1	1	1	0	1	1	7/8 (87.5)	low
Mamary, 2018 [32]	1	1	1	1	1	0	1	1	7/8 (87.5)	low
Mycroft, 2020 [33]	1	1	1	1	1	0	1	1	7/8 (87.5)	low
Tran, 2020 [34]	0	1	1	1	0	0	1	1	5/8 (62.5)	moderate
Jaen-Moreno, 2021 [35]	1	1	0	0	1	1	1	1	6/8 (75)	low
Stav, 2007 [36]	1	0	1	1	0	0	0	1	4/8 (50)	moderate
Yawn, 2009 [37]	1	1	1	1	1	0	0	1	6/8 (75)	low
Makinson, 2014 [38]	1	1	1	1	0	0	1	0	5/8 (62.5)	moderate
Lee, 2015 [43]	1	1	1	1	1	1	1	0	7/8 (87.5)	low
Sansores, 2015 [40]	1	1	1	1	1	1	1	1	8/8 (100)	low
Sansores, 2015 [40]	1	1	1	1	1	1	1	1	8/8 (100)	low
Labor, 2016 [41]	1	1	1	1	1	1	0	1	7/8 (87.5)	low
Su, 2019 [42]	0	1	1	1	1	1	0	1	6/8 (75)	low
Tran, 2020 [34]	0	1	1	1	0	0	1	1	5/8 (62.5)	moderate
Hwang, 2021 [43]	0	1	1	1	1	1	0	1	6/8 (75)	low
Yangui, 2021 [44]	1	1	1	1	1	1	0	1	7/8 (87.5)	low
Thorn, 2012 [45]	1	0	1	1	1	1	1	1	7/8 (87/5)	low
Ching, 2014 [46]	1	1	1	0	0	0	1	1	5/8 (62.5)	moderate
Kim, 2016 [47]	1	1	1	1	1	0	1	1	7/8 (87.5)	low
Korczyński, 2017 [48]	1	1	1	1	1	0	1	1	7/8 (87.5)	low
Liang, 2018 [49]	1	1	1	1	1	0	0	1	6/8 (75)	low
Lin, 2021 [50]	1	1	1	1	1	1	1	1	8/8 (100)	low

Table S3. Publications addressing the diagnostic screening of COPD along with the characterization of patient inclusion criteria.

	First author, year of publication	Type of spirometer	Inclusi on criteria: age; PY	Inclusion criteria (symptoms)	The diagnostic questionnaire used	% newly diagnosed COPD
1	Kotz, 2008 [25]	Portable	40–70; ≥ 10	at least one respiratory symptom	CDQ	41.1
2	Represas-Represas, 2016 [30]	Portable	> 40; ≥ 10	'symptoms suggestive of COPD'	N/A	31.5
3	Al Lami, 2017 [31]	Portable	> 35;> 20	LFQ ≤18	LFQ	16.7
4	Tran, 2020 [34]	Portable	> 40; > 10	'symptoms suggestive of COPD'	CAT; mMRC	27.2
5	Yawn, 2009 [37]	Conventional	> 40; ≥ 10	productive cough for at least 3 consecutive months in each of 2 successive years	MRC; modified ATS respiratory questionnaire	26
6	Lee, 2015 [39]	Conventional	≥ 75; ≥ 20	patients positive on the CTS screen to at least one question	COPD Screening Questions by CTS	36.4
7	Sansores, 2015 [40]	Conventional	> 40; ≥ 10	at least one respiratory symptom	'questionnaire containing 28 items'	11.4
8	Su, 2019 [42]	Conventional	≥ 40; ≥ 20	'subjects with respiratory symptoms'	CAT	47.9
9	Tran, 2020 [34]	Conventional	> 40; > 10	'symptoms suggestive of COPD'	CAT; mMRC	27.2
10	Hwang, 2021 [43]	Conventional	> 40; > 10	'subjects with respiratory symptoms'	mMRC	47.9
11	Kim, 2016 [47]	COPD-6 + conventional	> 40; > 10	'subjects with respiratory symptoms'	The"Could it be COPD?" questionnaire	23.7
12	Lin, 2021 [50]	Portable + conventional	≥ 40; ≥ 10	at least one respiratory symptom	CAT	27.8

CAT: the COPD Assessment Test; CTS: Canadian Thoracic Society; CDQ: the COPD Diagnostic Questionnaire; LFQ: the Lung Function Questionnaire, MRC: the Medical Research Council Dyspnea Scale; mMRC: the Modifed Medical Research Council Dyspnea Scale; ND: no data aplicable;
